# Efficacy of Vitamin D Supplementation in Accelerating Sputum Conversion of Bacteriologically Diagnosed Tuberculosis Patients: A Meta-Analysis

**DOI:** 10.7759/cureus.94668

**Published:** 2025-10-15

**Authors:** Maria-Angelica B Lerma, Ayla Niel L Castillo, Gelza Mae A Zabat

**Affiliations:** 1 Internal Medicine, Department of Medicine, St. Luke's Medical Center, Manila, PHL; 2 Internal Medicine, St. Luke's Medical Center, Quezon City, PHL; 3 Section of Infectious Diseases, St. Luke’s Medical Center, Taguig City, PHL; 4 Section of Infectious Diseases, St. Luke’s Medical Center, Quezon City, PHL

**Keywords:** adjunctive therapy, randomized controlled trials, sputum culture conversion, tuberculosis, vitamin d supplementation

## Abstract

Tuberculosis (TB) is a global pandemic disease whose first-line treatment is with a combination of four antimicrobials commonly abbreviated as HRZE (isoniazid, rifampin, pyrazinamide, and ethambutol). However, some consider the regimen inadequate, thus conducting numerous trials to search for adjunctive therapies, including vitamin D supplementation. Trials so far have led to mixed results. The present study aims to consolidate data on the effects of vitamin D supplementation on clinical outcomes, specifically rates of sputum culture conversion. PubMed databases were searched for peer-reviewed articles published in English between the years 2013 and 2023 that deal with human subjects, designed as randomized controlled trials (RCTs), utilizing vitamin D supplementation in addition to anti-TB therapy, with a measured outcome of the rate of sputum culture conversion at specific follow-up intervals. The risk of bias (RoB) was assessed using the Cochrane RoB 2 tool. Initially, 1,225 articles were found via database search, narrowed to six articles after screening and full-text reading. A significant difference was found between the vitamin D and control/placebo groups in rates of sputum culture conversion at eight weeks, with the intervention group having an increased odds of culture conversion by 1.63 (95% CI 1.13, 2.35, p < 0.01). The same was not found, however, at 16 weeks (OR = 0.99, 95% CI 0.71, 1.38, p > 0.05). Statistical heterogeneity was minimal, but the studies significantly differed in the dosing regimens of the intervention, populations included, and attrition rates. The RoB was also low, but there were concerns due to high attrition rates, lack of blinding, and the addition of unplanned post-hoc analysis. The beneficial effects of vitamin D supplementation, if any, involve eliciting an early response to treatment, reflected in improved culture conversion rates at earlier follow-up intervals. Possible factors mentioned in some studies that may lead to variations in the effect of vitamin D supplementation include vitamin D status (sufficiency/insufficiency/deficiency), presence of drug resistance, and genetic polymorphisms; these may be explored in future studies sufficiently powered to examine such a relationship.

## Introduction and background

Tuberculosis (TB) is a bacterial infection caused by *Mycobacterium tuberculosis *and is spread from person to person via droplets. It can affect any organ but affects the lungs in up to 75% of cases [1]. It is a global pandemic disease that, until the advent of the COVID-19 pandemic, was the leading cause of death from a single agent, ranking above HIV/AIDS [2]. Furthermore, the high prevalence of multidrug-resistant (MDR) and extensively drug-resistant (XDR) TB converges with the pandemics of HIV and diabetes mellitus (DM), with reports of increasing incidence in immunocompromised patients [3,4]. There was a noted drop in the reported number of newly diagnosed cases from 2019 to 2020, possibly due to underreporting and underdiagnosis, but recent figures have climbed back up to 6.4 million in 2021. The estimated number of deaths has likewise increased, from estimates of 1.4 million in 2019 and 1.5 million in 2020 to the most recent estimate of 1.6 million in 2021 [2]. Eight countries account for up to two-thirds of worldwide deaths, with the Philippines being counted among these eight [5].

Investigations for working up cases suspicious of TB include chest X-rays, CT scans, sputum microscopy, sputum cultures, and nucleic acid amplification tests. In the local setting, the Manual of Procedures of the National Tuberculosis Control Program states that rapid diagnostic tests (RDTs) such as Xpert MTB/RIF are the primary diagnostic test for both adults and children, with smear microscopy or loop-mediated isothermal amplification (LAMP) considered as alternative tests. First-line anti-tuberculous therapy (ATT) is with a combination of four antimicrobials - ethambutol, isoniazid, pyrazinamide, and rifampicin [1,6]. This regimen, however, has been deemed inadequate by some due to being long, and if cured, most patients are still left with bronchiectasis and fibrosis [7]. Due to issues with the current standard regimen and the high morbidity and mortality associated with the disease, several research works over the past years have thus aimed to identify supplemental or adjunct treatment. Such research mainly explores host-directed therapy, which involves modifying the function of host cells to counteract damage and complications caused by MTB [5]. One such article included in the current study explores, aside from vitamin D, dovramilast (CC-11050), a type 4 phosphodiesterase inhibitor; everolimus, a mTOR inhibitor; and auranofin, a gold salt that accumulates in macrophages [7]. Others mentioned in the literature include sodium 4-phenylbutyrate, pentoxifylline, GM-CSF, recombinant human interleukin, NAC, and Immunoxel, generally with favorable results [5].

Vitamin D is a micronutrient primarily involved in bone mineralization, but its role in various immune functions, including maintaining monocytes and macrophages, has also been elucidated [8,9]. Its major circulating metabolite, 1,25-hydroxyvitamin D (1,25[OH]D), supports innate antimicrobial immune responses, inducing gene expression of beta-defensin 2 and human cathelicidin LL-37, and autophagy in infected macrophages/monocytes, suggesting adjunctive vitamin D might enhance response to ATT [4]. Many epidemiologic studies have shown inverse relationships between vitamin D and the incidence of various infections and chronic diseases; there are associations between vitamin D deficiency and susceptibility to TB, risk of progression from infection to disease, conversion from latent to active infection, severity of disease, rates of relapse, and risk of contracting the disease by close contacts [4,9,10]. Deficiency may also accelerate CD4+ decline in HIV-positive patients, which is a possible mechanism by which the risk of infections is increased even in the HIV-negative population [1]. Such associations are alarming in light of reports that almost 50% of the world population may be afflicted by vitamin D deficiency, with an even higher rate in PTB patients at 87% [8]. Vitamin D supplementation is thus being explored as one of the possible adjunctive treatments during ATT, but this is not a new consideration; there has already been historical use of cod and other fish liver oils for the treatment of TB in the pre-antibiotic era [10-12].

Clinical trials have been conducted in recent years. However, the methods, dosage, and outcomes have varied substantially, with some trials showing that supplementation has no effect on outcomes and some showing an effect for specific populations, such as patients with polymorphisms of the vitamin D receptor (VDR) gene, MDR-TB cases, and patients with documented vitamin D deficiency [1,9,11]. In trials with positive results, proposed mechanisms by which vitamin D exerts its effect include upregulation of cathelicidin and LL-37, influencing CD4+ T cell activity, and enhancement of MTB antigen-induced IFN-γ secretion, resulting in intracellular killing of MTB and granuloma formation [1,9,10]. Consequently, in a country such as the Philippines with a high TB burden, there is a compelling need to investigate the benefits of vitamin D supplementation, particularly with regard to early sputum and culture conversion. Such investigations can be helpful in further optimizing the management of PTB. The studies conducted to investigate the effect of vitamin D in the management of PTB have shown inconsistent results, hence the conduct of the present study to determine whether vitamin D significantly accelerates the rate of sputum conversion.

This abstract was presented as a poster presentation at TBScience 2024, in Bali, Indonesia, on November 12-16, 2024.

## Review

Methodology

This meta-analysis was performed according to the PRISMA 2020 statement. A comprehensive systematic search of PubMed databases was performed from July to August 2023 to identify publications in the English language for relevant peer-reviewed articles with publication dates between 2013 and 2023. The following search criteria were used: ("vitamin d"[MeSH Terms] OR "vitamin d"[Title/Abstract] OR "calcifediol"[MeSH Terms] OR "calcidiol"[Title/Abstract] OR "calcifediol"[Title/Abstract] OR "1 25 hydroxyvitamin d"[Title/Abstract]) AND ("tuberculosis"[MeSH Terms] OR "tuberculosis"[Title/Abstract] OR "lung tuberculosis"[Title/Abstract] OR "pulmonary tuberculosis"[Title/Abstract] OR "ptb"[Title/Abstract]). Criteria for inclusion included the following: (1) papers published between 2013 and 2023, (2) randomized clinical trials (RCTs), (3) use of vitamin D supplementation during ATT, (4) explored the outcomes of sputum culture conversion, and (5) dealt with human subjects. Articles found in the initial search were screened through their titles and abstracts. Irrelevant papers were excluded, while the rest were chosen for full-text reading. From there, articles with insufficient data were further screened out. Criteria for exclusion in the current meta-analysis included letters, editorials, book chapters, systematic reviews, other meta-analyses, prospective or ongoing trials, RCTs without data on sputum smear or culture conversion, and research papers not published in the English language. Only published studies on patients above 18 years old and newly diagnosed with pulmonary TB (as determined by symptoms and signs, sputum smear/culture findings, and Xpert MTB/RIF results) were included. The methodology of each study was reviewed to confirm the target population. Investigation of outcomes regarding sputum conversion was limited to the effect of vitamin D supplementation on rates of sputum culture conversion at specific time points. Studies measuring rates of sputum smear conversion and time to sputum smear/culture conversion were excluded.

Selection of Studies

Two authors (MBL and ALC) independently screened and reviewed the abstracts and articles for inclusion. Articles were selected based on the inclusion criteria, and the decision to include the article was made through consensus. An initial database search with the search criteria yielded 1,225 publications. After refining the search parameters, excluding papers outside the publication date range and studies that were not RCTs, the search yielded 28 articles. Based on the title and abstract, a further 15 articles were found to have outcomes or study designs not compatible with the inclusion criteria, and these were excluded. Full texts of 13 studies were reviewed, and of these, three were found to have the concomitant use of other therapies, while four explored other outcomes related to sputum conversion but did not fit the criteria. Thus, six articles were ultimately eligible for meta-analysis. Studies with comparison groups and outcomes not compatible with the goals of this review were excluded.

Data Extraction and Risk-of-Bias Assessment 

Selected articles were downloaded and independently reviewed by the reviewers. Discrepancies in the selection process were resolved through discussion and reaching a consensus. Consultation with a third expert investigator was done when a consensus could not be reached. A data collection form was created to extract information from each article, including the study's authors, year of publication, sample size, recruitment period, criteria for inclusion/exclusion with a focus on documentation of TB infection and factors significantly affecting the current outcome, intervention and placebo/control, duration of follow-up, and clinical outcomes measured not limited to rates of sputum culture conversion at specific time points. Characteristics of the patient populations at baseline were also extracted and formatted into a separate table. Quality assessment was done using the Cochrane Risk of Bias (RoB) 2 tool. Each article was critically appraised for each domain of bias: bias from the randomization process, bias due to deviations from intended interventions, bias due to missing outcome data, bias in the measurement of the outcome, and bias in the selection of the reported result. These were graded as high, low, or with some concerns, and discrepancies were settled through constructive discussion and reaching a consensus. The overall RoB in each article was then decided based on the grading of each section.

Data Synthesis and Analysis 

Meta-analysis was performed only for those outcomes in which data from at least two studies were present. Following these criteria, analysis was performed only for the culture conversion rate measured at eight and 16 weeks. Data were also present for rates of culture conversion at four, 20, and 24 weeks. However, for each of the latter cases, only one article could provide usable data. Statistics were computed using Cochrane Review Manager (RevMan) 5 using the Mantel-Haenszel approach and a random-effect model, presenting the output as odds ratios for dichotomous data. Statistical significance was pegged at p = 0.05. Statistical heterogeneity was estimated using Chi-square and Higgins I^2^ statistic, with values classified as minimal, moderate, substantial, and considerable for scores of 0-40%, 30- 60%, 50-90%, and 75-100%, respectively, as patterned from the values used in the Cochrane Handbook for Systematic Review of Interventions. For instances where values were presented as median and first and third quartiles, estimates of mean and standard deviation were obtained using the methods presented by McGrath et al. (2020), which perform well for non-normal and skewed data. In cases where the first and third quartiles are missing and only the IQR is presented, a rough estimate of the standard deviation is made by dividing the IQR by 1.35.

An initial search using the search criteria previously outlined in the methodology yielded 1,225 articles, which were reduced to 28 once the criteria for inclusion in the present study were applied. Manual screening of titles and abstracts resulted in the exclusion of several articles due to having outcomes unrelated to the present study or study designs incompatible with the analysis. The last phase of screening via reading the full texts resulted in the exclusion of four articles due to having outcomes related to sputum conversion but not specifically to rates of sputum culture conversion, and three articles due to the concomitant use of other therapies (namely, vitamin A, L-arginine, and phenylbutyrate) alongside vitamin D supplementation. The design of the latter studies was such that outcomes were only present for groups using both vitamin D and another treatment modality. Thus, the specific effect of vitamin D could not be isolated. Data were still extracted from these articles for the review and discussion but were excluded from the meta-analysis.

**Figure 1:**
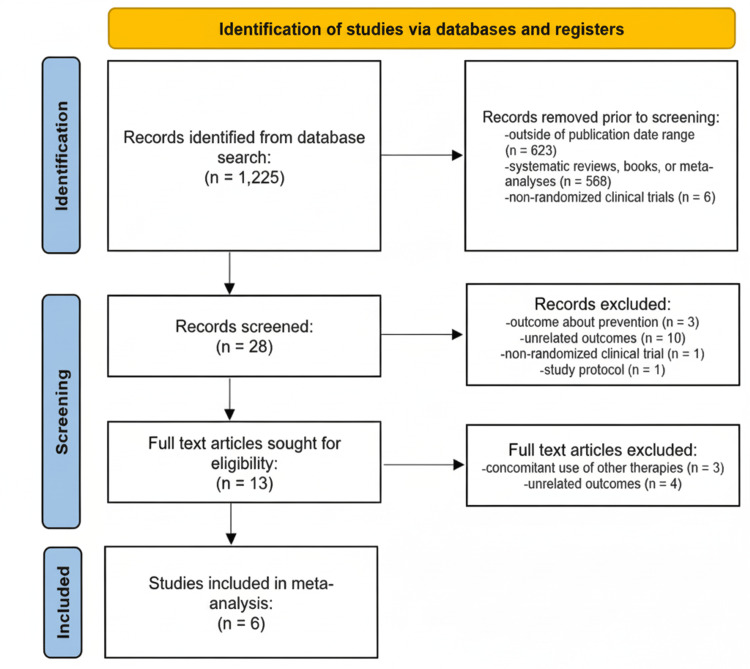
Flow chart of the article selection, screening, and inclusion

Figure 1 illustrates the process of searching, screening, and including articles for the final meta-analyses. The process resulted in a final count of six studies included in the meta-analyses. Many of these have multiple outcomes published, but the outcomes being explored in the present study only include the rate of culture conversion at eight weeks (n = 6) and the rate of culture conversion at 16 weeks (n = 2). The characteristics of the studies included in the meta-analysis are tabulated in Table 1, outlining their methodologies and the outcomes explored by each aside from the ones currently being studied.

**Table 1:** Characteristics of studies on the effect of vitamin D supplementation on sputum conversion n: sample size of participants, BMI: body mass index, HIV: human immunodeficiency virus, TB: tuberculosis, PTB: pulmonary tuberculosis, EPTB: extrapulmonary tuberculosis, Mtb: *Mycobacterium tuberculosis*, ATT: anti-tuberculosis therapy, MDR-TB: multidrug-resistant tuberculosis, Xpert MTB/RIF: Xpert Mycobacterium tuberculosis/rifampicin, AFB: acid-fast bacillus, PO: per orem/by mouth, OD: once a day, IU: international units, wks: weeks, q2 weeks: every two weeks, vit D₂: ergocalciferol, vit D₃: cholecalciferol 25(OH)D = 25-hydroxyvitamin D, CRP: C-reactive protein, ESR: erythrocyte sedimentation rate, FEV1: forced expiratory volume in one second, FVC: forced vital capacity, WBC: white blood cell count, PMN: polymorphonuclear leukocytes, MUAC: mid-upper arm circumference, LL-37: cathelicidin Hasanain AF, et al. (2018) [3], Mily A et al. (2015) [4], Wallis R et al. (2021) [7], Tukvadze N et al. (2015) [9], Daley P et al. (2015) [11], Ganmaa D et al. (2017) [12]

Authors	Location and recruitment period	No. of participants	Criteria inclusion/exclusion	Intervention	Placebo/ control	Followup period	Clinical outcome(s)
Daley et al., 2015 [11]	India, 2010-2011	n = 289	- At least one documented positive sputum smear - One dose of TB treatment or fewer - Excluded HIV and MDR TB	100,000 IU of vitamin D₃ (2.5 mg), PO, q2 weeks x 4 doses	Miglyol oil	Six months	- Time to culture and smear conversion - Culture and smear conversion rate - Karnofsky performance status - BMI - 25(OH)D concentration, calcium levels
Mily et al., 2015 [4]	Bangladesh, 2010-2013	n = 288	- Newly diagnosed sputum smear positive PTB - Excluded HIV and relapse TB	5,000 IU of vitamin D₃ oil, PO, OD	Tablet bulking agent/ Miglyol oil	24 weeks	- Culture conversion rate - Time to smear conversion - Cough and fever remission - Weight gain - Radiological findings - 25(OH)D concentration, calcium levels - LL-37 expression, intracellular Mtb killing
Tukvadze et al., 2015 [9]	Georgia, 2008-2014	n = 199	- Newly diagnosed PTB via signs and symptoms and positive AFB smear (later confirmed with culture) - <=7 days ATT - Excluded relapse TB, EPTB, and known MDR-TB	50,000 IU of vitamin D₃ (1.25 mg), PO, x 8 weeks, then q2 wks x 8 more weeks	Placebo	15 weeks	- Time to culture conversion - Culture conversion rate - 25(OH)D concentration
Ganmaa et al., 2017 [12]	Mongolia, 2014-2014	n = 390	- Newly diagnosed PTB and ATB visible on smear microscopy - <=7 days ATT - Excluded HIV	140,000 IU of vitamin D₃ (3.5 mg), PO, q2 weeks x 4 doses	Placebo	Eight weeks	- Culture conversion rate - Radiological findings - 25(OH)D concentration, corrected calcium - CRP, albumin, mean ESR, total WBC, PMN, monocyte count - BMI, MUAC, triceps skin fold thickness, scapular skin fold thickness
Hasanain et al., 2018 [3]	Egypt, 2014-2017	n = 496	- Newly diagnosed PTB via positive sputum culture - Excluded previous ATT, EPTB, HIV	600 IU of vitamin D₃, PO, OD	Anti-TB treatment only	Five months	- Culture conversion rate - Liver function tests
Wallis et al., 2021 [7]	South Africa, 2016-2018	n = 80	- Positive Xpert MTB/RIF with rifampicin-susceptible MTB - Excluded previous TB, HIV	5 mg of vitamin D₂ on day 1, 2.5 mg on days 28 and 56	Anti-TB treatment only	Six months	- Culture conversion rate - Adverse events - FEV1, FVC

The RoB assessment of the included articles stratified by domains of possible sources of bias is summarized in Table 2.

**Table 2:** Summary of risk of bias according to possible sources of bias as measured by the Cochrane Risk of Bias 2 Tool 🟢 Low risk of bias: The study appears to have addressed this potential source of error adequately. 🟡 Unclear risk of bias: The study did not provide enough information to make a clear judgment. Hasanain AF et al. (2018) [3], Mily A et al. (2015) [4], Wallis R et al. (2021) [7], Tukvadze N et al. (2015) [9], Daley P et al. (2015) [11], Ganmaa D et al. (2017) [12]

Study	Random sequence generation	Allocation concealment	Blinding of participants and personnel	Blinding of outcome assessment	Incomplete outcome data	Selective reporting	Other bias
Daley et al., 2015 [11]	🟢	🟢	🟢	🟡	🟢	🟢	🟢
Ganmaa et al., 2017 [12]	🟢	🟢	🟢	🟢	🟢	🟢	🟢
Hasanain et al., 2018 [3]	🟢	🟢	🟢	🟢	🟢	🟢	🟢
Mily et al., 2015 [4]	🟢	🟢	🟢	🟢	🟡	🟢	🟢
Tukvadze et al., 2015 [9]	🟢	🟢	🟢	🟢	🟡	🟢	🟢
Wallis et al., 2021 [7]	🟢	🟢	🟢	🟢	🟢	🟡	🟢

Results

**Figure 2:**
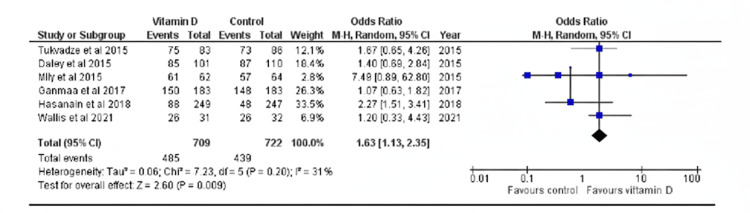
Forest plot of the rate of sputum culture conversion in vitamin D supplementation versus control groups at eight weeks Hasanain AF et al. (2018) [3], Mily A et al. (2015) [4], Wallis R et al. (2021) [7], Tukvadze N et al. (2015) [9], Daley P et al. (2015) [11], Ganmaa D et al. (2017) [12]

The RoB is generally low for all studies, but possible issues were found about missing outcome data, lack of blinding of both participants and facilitators/investigators in select studies, and the addition of post-hoc analysis once results had been collated. Attrition rates and percentages of missing data were high in most studies, reaching up to a quarter of the total samples in one, but this was offset by the initial computations in sample size accounting for losses in data and the use of intention-to-treat or modified intention-to-treat analysis. The lack of blinding was also a significant factor, but due to the objectivity in the measurement of the clinical outcomes currently being explored, the ultimate effect on the results was deemed to be minimal. A significant difference was found between the vitamin D and control/placebo groups in rates of sputum culture conversion at eight weeks, with the intervention group having an increased odds of culture conversion by 1.63 (95% CI 1.13, 2.35, p < 0.01) (Figure 2).

**Figure 3:**
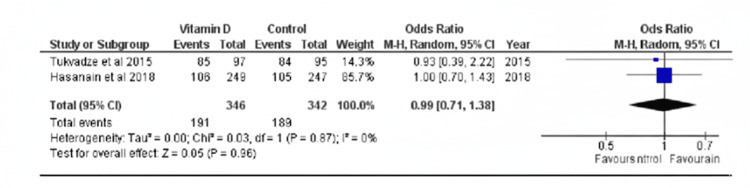
Forest plot of rate of sputum culture conversion in vitamin D supplementation versus control groups at 16 weeks Hasanain AF et al. (2018) [3], Mily A et al. (2015) [4], Wallis R et al. (2021) [7], Tukvadze N et al. (2015) [9], Daley P et al. (2015) [11], Ganmaa D et al. (2017) [12]

The same was not found, however, at 16 weeks (OR = 0.99, 95% CI 0.71, 1.38, p > 0.05). The first article showed minimal to moderate but non-significant heterogeneity, while the second had minimal and non-significant heterogeneity (p > 0.05, I^2^ = 31%; p > 0.05, I^2^ = 0%, respectively). Possible sources of heterogeneity between the studies include the following: differences in the dosing regimens, including formulation, the total amount of IU administered, frequency of intake, and duration of the intervention; the decision to exclude/include specific populations such as MDR-TB, EPTB, and relapse TB patients in some studies; and differences in attrition rates, as shown in Figure 3.

Discussion

The present study aimed to demonstrate the effect of vitamin D supplementation on sputum culture conversion among groups with vitamin D supplementation and those who received only the standard ATT. Meta-analysis showed a slightly better outcome for the vitamin D group at eight weeks, but the same was not reproduced at 16 weeks. This is in line with the conclusion of several articles, stating that the benefits of vitamin D may be limited to only speeding up sputum conversion, reflected as higher rates of conversion at earlier follow-up intervals; total cure rates are less likely to be improved [1]. Moreover, several factors have been suggested by the published literature as essential considerations in future trials and research due to differential effects.

Several studies investigated and recorded vitamin D levels [2-4], noting that most TB patients at baseline had levels that corresponded to deficiency or were below the threshold of sufficiency. However, the criteria that they used for categorization varied. Sinha et al. [5] postulated that only deficient patients may show improvement in outcomes due to the need for a critical level of vitamin D for the optimal activation of IFN-γ secretion. Variability in outcomes may thus be affected by the unequal distribution of patients with differing categories of vitamin D levels in control and intervention groups. Such a hypothesis could not be tackled in the present study due to the lack of stratification or subgrouping of data but is supported by the methodology of Wen et al. [1], where participants were stratified into sufficient and deficient groups, with a subset of the deficient patients receiving the supplementation regimen. Outcomes were significantly more favorable in the intervention group than their deficient counterparts who did not receive treatment but were not significant compared to the vitamin D-sufficient control group.

Polymorphisms of the VDR gene are ethnically divergent and are another hypothesized cause of variation in the degree of benefit from vitamin D supplementation between populations from different geographical locations [2]. Although older studies previously reported more significant effects on outcomes for patients with specific VDR genotypes, the trials included in the present study have shown no differences in the effects of supplementation on sputum smear/culture conversion in patients with differing polymorphisms. However, Mily et al. [4] identified novel variants in VDR and CYP27B1 that may serve as points of interest for future research [3]. The former paper further states that the negative findings may be due to the underpowered trials to explore such interactions.

Data on MDR-TB patients is only available from two studies due to it being part of the exclusion criteria of some studies and a need for separate reporting of data for this population in others. Hasanain et al. [3] and Mily et al. [4] reported substantial but insignificant trends favoring vitamin D supplementation in MDR-TB patients. Higher culture conversion rates were observed, possibly due to endogenous immune-stimulating effects contributing to increased sputum clearance [6]. However, as with VDR polymorphisms, future trials will have to be conducted, which are specifically powered to explore this association.

Overall, the results suggest that the beneficial effects of vitamin D supplementation, if any, involve eliciting an early response to treatment, reflected in improved culture conversion rates at earlier follow-up intervals. Important mediators of this effect that have been previously tackled but only in passing include vitamin D levels and degree of sufficiency, genetic polymorphisms, and antibiotic resistance, specifically classification as MDR TB.

One of the significant limitations of the study is the need for more consistency between studies in their methodologies. Dosages and administration schedules of vitamin D3 supplementation vary between the papers included. Although there were no or minimal significant differences between intervention and control groups of the individual papers, inclusion/exclusion criteria differed. Thus, the inclusion of specific populations that may have affected the results, such as MDR-TB patients and individuals with specific genetic polymorphisms.

## Conclusions

Vitamin D supplementation may play an essential part as an adjunct in treating TB. Results point to a possible acceleration of the rate of sputum culture conversion, supported by higher rates of conversion at earlier points of follow-up. However, the variability of results in currently published trials makes it difficult to draw conclusive statements. Cure rates were found to be ultimately unaffected. Thus, the beneficial effects of vitamin D are limited to hastening treatment response. Further studies with standardized methodologies, especially about patient inclusion criteria, vitamin D supplementation regimens, duration and time points of follow-up, and measurement of outcomes, will have to be conducted. The effects of covariates such as sex, vitamin D status at baseline, presence of drug-resistant TB, and genetic polymorphisms will likewise have to be investigated and controlled for, in trials sufficiently powered to explore such interactions.
